# Two Native *Anagrus* spp. (Hymenoptera: Mymaridae) Are Egg Parasitoids of the Invasive Two-Spot Cotton Leafhopper *Amrasca biguttula* (Ishida) (Hemiptera: Cicadellidae) in Florida, USA

**DOI:** 10.3390/insects17030269

**Published:** 2026-03-03

**Authors:** Alexandra M. Revynthi, Serguei V. Triapitsyn, Yisell Velazquez-Hernandez, Paul F. Rugman-Jones

**Affiliations:** 1 Tropical Research and Education Center, Institute of Food and Agricultural Sciences, University of Florida, Homestead, FL 33031, USA; 2 Department of Entomology, University of California, Riverside, CA 92521, USApaulrj@ucr.edu (P.F.R.-J.)

**Keywords:** natural enemy, invasive pest, biological control, cotton jassid, fairyfly

## Abstract

The two-spot cotton leafhopper, *Amrasca biguttula* (Ishida) (Hemiptera: Cicadellidae), was recently detected in Florida and other southeastern states, USA. This is a quarantine pest of regulatory significance, since it can infest staple crops, such as okra, cotton, eggplant, and tropical hibiscus. While collecting infested okra plants in Homestead, Florida, five female *Anagrus* (Hymenoptera: Mymaridae) parasitoids emerged from eggs of *Am. biguttula*. The specimens were identified morphologically and molecularly by sequencing the cytochrome c oxidase subunit I (COI) gene and internal transcribed spacer 2 (ITS2) region of nuclear ribosomal RNA. Two *Anagrus* species were identified: *Anagrus vulneratus* and *Anagrus* sp. near *vulneratus*. These parasitoids are not known to occur in the Old World, the origin of *Am. biguttula*. Rather, they are native to North America. The available evidence suggests that the collected specimens switched from unknown local hosts in southern Florida to parasitize eggs of the invasive *Am. biguttula*. Future research to ascertain the identity of *A.* sp. near *vulneratus* and evaluate the efficiency of both parasitoids as natural enemies of *Am. biguttula* is warranted.

## Simple Summary

The two-spot cotton leafhopper, *Amrasca biguttula* (Ishida) (Hemiptera: Cicadellidae), is a regulated invasive pest recently detected in Florida and other southeastern states, USA. This insect attacks staple crops, such as okra, cotton, eggplant, and tropical hibiscus. While collecting infested okra plants in Homestead, Florida, one female *Anagrus* (Hymenoptera: Mymaridae) parasitoid was collected using a brush, whereas five parasitoids emerged from *Am. biguttula* eggs. The parasitoids were identified morphologically and molecularly as *Anagrus vulneratus* and *Anagrus* sp. near *vulneratus*. These parasitoids are native to North America and likely switched from unknown local hosts in southern Florida to parasitize *Am. biguttula* eggs. Future research is warranted to evaluate the efficiency of both parasitoids as natural enemies of *Am. biguttula*.

## 1. Introduction

The two-spot cotton leafhopper *Amrasca biguttula* (Ishida) (Hemiptera: Cicadellidae), also known under the common names two-spotted cotton leafhopper, cotton jassid, and okra leafhopper, is a serious pest of okra (*Abelmoschus esculentus* (L.) Moench), cotton (*Gossypium hirsutum* L.), eggplant (*Solanum melongena* L.), sunflower (*Helianthus annuus* L.), roselle (*Hibiscus sabdariffa* Rottler), and tropical hibiscus (*Hibiscus rosa-sinensis* L.) [1,2]. Both nymphs and adults feed on the underside of leaves. Using the piercing-sucking mouthparts, the leafhopper causes significant damage to the plants. The symptoms include leaf yellowing and curling, “hopperburn” (necrosis and drying of leaf margins), stunted growth, and crookedness of okra pods. In cotton, yield losses range from 19 to 49% [3,4], while in okra and eggplant, losses may reach up to 50% and 37%, respectively [5]. This pest is currently not known to vector any plant pathogens [1].

*Amrasca biguttula* is native to Asia and Oceania and has been reported from Afghanistan, Australia, Bangladesh, China, Christmas Island, French Polynesia, Guam, Iran, India, Indonesia, Japan, Laos, Malaysia, Myanmar, Nepal, Northern Mariana Islands, Pakistan, Philippines, Sri Lanka, Taiwan, Thailand, and Vietnam [6,7]. However, it is adventive in Iraq, West Africa, and the Caribbean [8,9], including Puerto Rico, the US Virgin Islands, Barbados, Antigua, Anguilla, and St. Kitts and Nevis [10], as well as in parts of Central America, where it has been confirmed at least in Honduras. Its recent detection in Florida in December of 2024 [10], in addition to its rapid spread in the Caribbean and southeastern USA [11], has raised concerns about its potential impact on staple American crops such as cotton, okra, and eggplant. In addition, a recent risk analysis highlights that Southwest Asia, sub-Saharan Africa, and South America are at high threat of invasion by *Am. biguttula* [12]. Moreover, rising temperatures are predicted to facilitate the pest’s establishment in other geographies.

Currently, *Am. biguttula* is considered a quarantinable pest of regulatory significance in the USA. In Florida, this pest is regulated by the Florida Department of Agriculture and Consumer Services-Division of Plant Industry (FDACS-DPI). Tropical hibiscus is an ornamental host plant intensively produced in Florida and shipped throughout the country; therefore, nurseries found with the leafhopper are being placed under “Stop Sale and Hold Order”. It is imperative to implement effective integrated pest management (IPM) programs to mitigate the economic damage caused by *Am. biguttula*. Notably, IPM programs developed should not rely on chemical control because there are reports of *Am. biguttula* resistance to pesticides across multiple insecticide classes [13]. Studies document moderate-to-very high resistance ratios to neonicotinoids in several cotton-growing regions, and recent work has demonstrated dimethoate (organophosphate, 1B) resistance with cross-resistance to some pyrethroids (3A) and novel modes of action [14,15,16]. Chemical control of *Am. biguttula* has relied heavily on systemic neonicotinoids (4A) (imidacloprid, thiamethoxam, acetamiprid) and, more recently, on newer chemistries such as dinotefuran, flonicamid and flupyradifurone, which generally show high field efficacy and rapid knockdown when applied as foliar sprays or seed treatments [17,18,19,20].

Natural enemies can play a pivotal role in regulating *Am. biguttula* populations. Fairyfly (Hymenoptera: Mymaridae) egg parasitoids have been repeatedly identified as the most important natural enemies attacking concealed *Am. biguttula* eggs, with higher parasitism rates observed on unsprayed or pesticide-free farms and clear reductions in leafhopper populations where parasitoids are abundant [21,22,23]. Several species of the genus *Anagrus* Haliday have been found to parasitize *Am. biguttula* eggs in Japan. Examples include *Anagrus atomus* (L.), *Anagrus japonicus* Sahad and *Anagrus turpanicus* Triapitsyn and Hu [22,23,24]. Adult *Am. biguttula* females insert their eggs into the veins of tender plant tissue [25]. The female *Anagrus* spp. parasitoids use the opening made by *Am. biguttula* to oviposit in the leafhopper eggs [26]. Within the family Mymaridae, *Arescon enocki* (Subba Rao and Kaur) and *Stethynium empoascae* Subba Rao have also been identified as egg parasitoids of *Am. biguttula* [23,24,27].

This study reports two *Anagrus* spp. for the first time in Florida and the entire New World associated with *Am. biguttula.* Here we present information on their morphological and molecular identification and discuss potential implications for the biological control of the invasive *Am. biguttula*.

## 2. Materials and Methods

### 2.1. Specimen Collection

**Figure 1:**
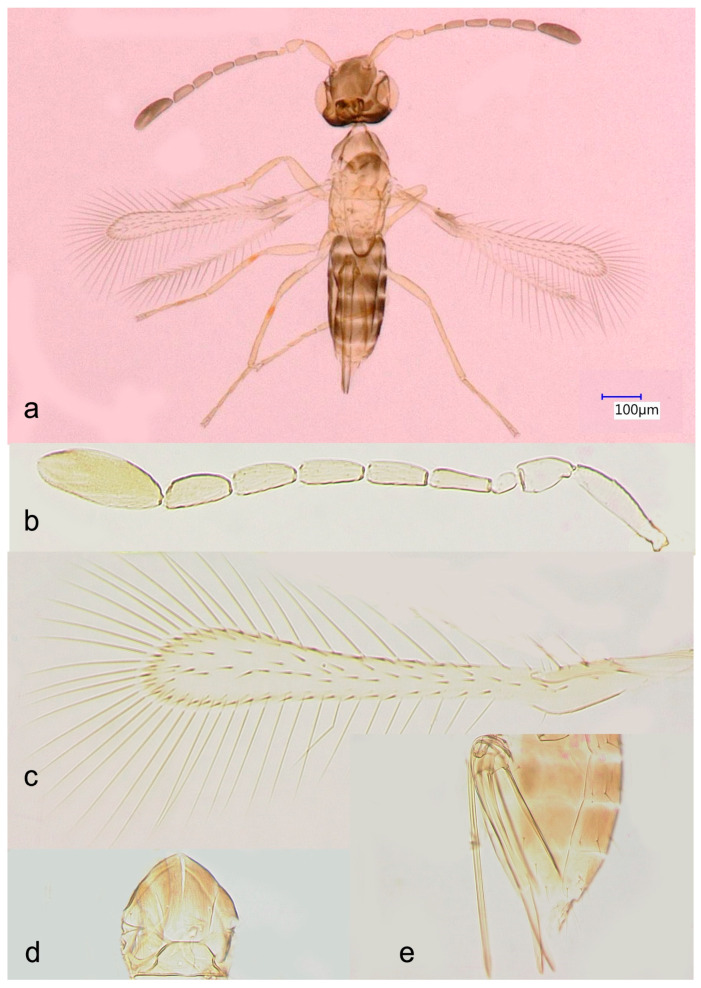
*Anagrus vulneratus* from Homestead, FL, USA (female): (**a**), habitus; (**b**), antenna (length: 458 μm); (**c**), fore wing (length: 470 μm); (**d**), mesoscutum and scutellum (length: 111 μm); (**e**), ovipositor (length: 246 μm).

On September 23, 2025, okra leaves infested with *Am. biguttula* nymphs and adults were collected from a 0.2 ha field at the Tropical Research and Education Center (TREC) of the University of Florida in Homestead (25°30′36″ N 80°30′22″ W, 2 m). No other leafhoppers were present on the okra plants. Leaves were inspected under a stereomicroscope (MZ6, Leica, Wetzlar, Germany) and one adult female parasitoid was collected using a fine brush (Winsor & Newton Cotman Series 111 Round #0000, Sheffield, UK). The specimen (Figure 1a) was slide-mounted using Hoyer’s medium [28] and kept at the Ornamental Entomology & Acarology laboratory at TREC.

Subsequent okra leaf collections were held on September 29, October 2, 14, 29, and November 4, 2025. Leafhoppers were removed from the okra leaves, which were then placed with the abaxial side facing up in a Petri dish (52 × 37 mm) (Dynalon, New York, NY, USA) with 1% agar (Fisher Chemical A360500 Agar CAS 9002-18-0 Powder, Fisherbrand, Pittsburgh, PA, USA). To ensure ventilation, the Petri dish lids had two holes (21 mm diameter) covered with a fine mesh (100 μm diameter). To simulate the natural leaf position on the plant, the Petri dishes were placed upside-down inside an incubator (Percival I-36LL, Percival Geneva Scientific, Williams Bay, WI, USA) at 27 ± 1 °C, 12:12 h L:D, and 70% RH. The leaves were inspected for parasitoid emergence every 24 h for a total period of seven days. Emerging parasitoids from okra leaves were kept in the Petri dish for 24 h for observation, after which they were transferred to 4 mL glass vials (Wheaton, DWK Life Sciences, LLC., Millville, NJ, USA) containing 95% ethanol. In total, ten okra leaves were incubated per sampling date, and five adult female parasitoids were collected during October 2, 14, 29, 2025. Since leafhoppers insert their eggs in the leaves, it was not possible to count the number of laid and parasitized eggs.

### 2.2. Taxonomic Identification

The five ethanol-preserved female specimens of *Anagrus* sp. (at that point we presumed that they were all conspecific) were shipped to the Entomology Research Museum, Department of Entomology, University of California at Riverside (UCRC) for further identification to species level. DNA was extracted by P. F. Rugman-Jones from all five individuals using a nondestructive method described below. Consequently, these primary molecular vouchers (under P. F. Rugman-Jones’ numbers PR25-794, PR25-795, PR25-796, PR25-797, and PR25-798), each of which was also assigned a unique identifier for its repository (UCRC ENT number), were slide-mounted in Canada balsam to be used for morphological identification.

A tentative taxonomic identification was made based on the obtained molecular data and using the morphological descriptions and keys in Triapitsyn [29,30,31] and Triapitsyn et al. [32]. The morphological terms of Triapitsyn [31] were used, with the following abbreviations: F—funicle segment of the female antenna or flagellomere of the male antenna; mps—multiporous plate sensillum or sensilla on the antennal flagellar segments (=longitudinal sensillum or sensilla, or sensory ridge(s)).

Slide-mounted specimens of *Anagrus* spp. were examined under a Zeiss Axioskop 2 plus compound microscope (Carl Zeiss Microscopy, LLC., Thornwood, NY, USA) and photographed using the Auto-Montage^®^ system (Syncroscopy, Princeton, NJ, USA). Photographs were retouched where necessary using Adobe Photoshop^®^ (Adobe Systems, Inc., San Jose, CA, USA).

### 2.3. DNA Extraction, Amplification, and Sequencing

DNA was extracted from five individual specimens using the HotSHOT method of Truett et al. [33] in a total volume of 80 µL. Using the polymerase chain reaction (PCR), a section of the “barcoding” region of the mitochondrial cytochrome c oxidase subunit I (COI) gene was amplified using the primers C1-J-1718 and C1-N-2191, or LCO1490 and HCO2198, as described in Triapitsyn et al. [34]. In a separate PCR, the internal transcribed spacer 2 (ITS2) region of nuclear ribosomal RNA (rRNA) was also amplified as described in Triapitsyn et al. [35]. Amplification was confirmed by gel electrophoresis, after which the amplicons were purified using the PCR Product Pre-Sequencing Kit (Applied Biosystems, Foster City, CA, USA) and directly Sanger-sequenced in both directions at the Institute for Integrative Genome Biology, University of California at Riverside. The parity of forward and reverse reads was checked, and we initially used BLASTn searches (online version BLAST+ 2.17.0) to compare our sequences with records in two complementary public repositories to obtain molecular support/guidance for their identification. Sequences of the ITS2 were compared with those in the comprehensive GenBank library, while COI sequences were compared with those in the more focused Barcode of Life Data System (BOLD). Based on the outcome of these searches, specimens of two further species for which COI sequences were not already available were sequenced. These included specimens of *A. vulneratus* Triapitsyn originally collected in Grand Junction, CO, USA, and an undetermined species that we previously referred to as *A.* sp. near *vulneratus* originally collected in Sonora, Mexico [32,36] (misidentified as *A. epos* Girault). Respective DNA vouchers from Triapitsyn et al. [32] were retrieved from storage (*A. vulneratus*, PR08-004 and PR08-192; *A.* sp. near *vulneratus*, PR09-038) and several fresh extractions were made of specimens from each of the original collections. These included two male *A.* sp. near *vulneratus* (PR25-799 and PR25-800), and one male (PR25-801) and one female (PR25-802) *A. vulneratus*, from the same collections in Colorado and Sonora, respectively, as reported in Triapitsyn et al. [32]. We subsequently amplified and sequenced COI for all these specimens. The ITS2 was also sequenced for each of the fresh extractions to confirm conspecificity with the earlier material. All sequences generated herein were deposited in GenBank (accession numbers PX978890-900, COI; PX983467-475, ITS2).

### 2.4. Genetic Analysis

The COI sequences of the five Florida specimens were combined with those from additional authoritatively identified (SVT) *Anagrus* spp., produced herein or garnered from earlier studies [35,37,38]. The combined sequences were aligned in MAFFT version 7.050 [39] and trimmed to a uniform length, delimited at the 5′ end by the site of the C1-J-1718 primer, and at the 3′ end by the removal of a ~50 bp nucleotide-deficient overhanging region. The result was a final sequence data matrix containing 17 terminal taxa, 396 nucleotide positions, and no gaps. Although not the full length (~658 bp) barcoding region, shortened barcodes have previously shown their utility for discriminating between the closely related species of other *Anagrus* complexes [40]. Genetic variation among the sequences was estimated by calculating uncorrected p-distances between all possible sequence pairs, using MEGA version 12 [41]. A neighbor-joining (NJ) tree based on the p-distances was constructed and branch support was estimated using a bootstrap procedure with 1000 replicates. The outgroup *A. atomus* was used to root the tree.

## 3. Results

### 3.1. Taxonomic Identification of the Two Anagrus Species from Florida

***Anagrus vulneratus* Triapitsyn** (Figure 1).

*Anagrus vulneratus* Triapitsyn in Triapitsyn et al. [32]: 9–12. Type locality: 39°02′31″ N 108°27′58″ W, 1450 m, Colorado State University Western Colorado Research Center—Orchard Mesa, Grand Junction, Mesa Co., CO, USA, from eggs of *Erasmoneura vulnerata* (Fitch) (Hemiptera: Cicadellidae) on cultivated grapes.

Material examined. USA: Florida, Miami-Dade Co., Homestead, UF/IFAS Tropical Research and Education Center, 25°30′36″ N 80°30′22″ W, 2 m, Y. Velazquez-Hernandez, on okra leaves infested with *Am. biguttula*: 23.ix.2025 (1 female, TREC); x.2025 (4 females, UCRC [PR25-795–798; UCRC ENT 573131, 283307, 573459, 573323, respectively]).

**Figure 2:**
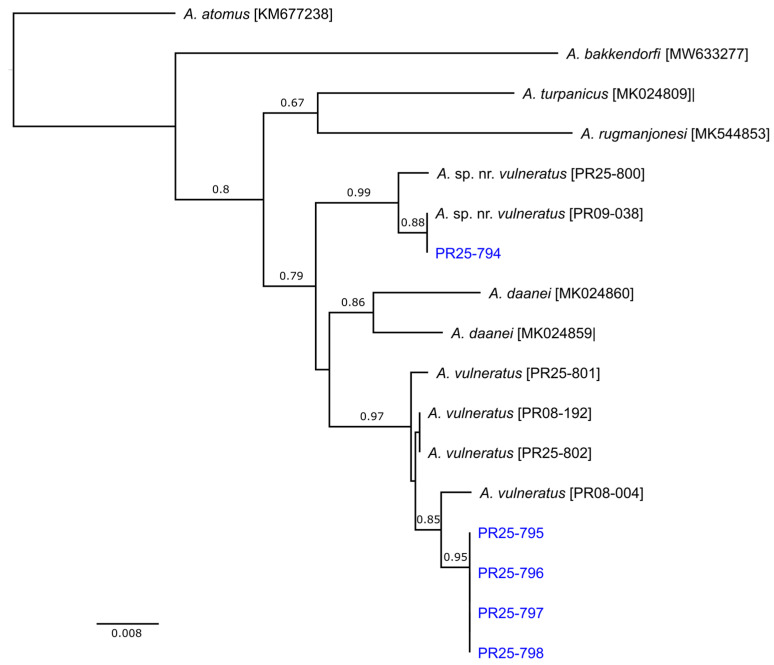
Relationships among the sequences of the cytochrome c oxidase subunit I (COI) gene from *Anagrus* species. Optimal unrooted NJ tree with the sum of branch length = 0.247. The percentage of replicate trees in which the associated taxa clustered together in the bootstrap test (1000 replicates) are shown next to the branches and the tree is drawn to scale, with branch lengths indicating uncorrected p-distance. Specimens collected from Florida are in blue.

Diagnosis: Molecularly, four of the five sequenced specimens from Florida match those of *A. vulneratus* from Colorado (Figure 2). This species belongs to the *incarnatus* species group of the nominate subgenus of *Anagrus*, as defined by Triapitsyn [31], and, based on the presence of a pair of adnotaular setae on the midlobe of the mesoscutum (Figure 1d) and a bare area on the widest part of the fore wing (Figure 1c), to the *A. epos* species complex. Triapitsyn et al. [32] keyed the species in this complex, in which they included the Nearctic *A. daanei* Triapitsyn, *A. epos* Girault, *A. tretiakovae* Triapitsyn, and *A. vulneratus* Triapitsyn. Triapitsyn et al. [32] also indicated that the Nearctic and Neotropical species, *A. empoascae* Dozier, is morphologically somewhat similar to these taxa in the complex, but molecular data for *A. empoascae* was not available. Ecologically, among Cicadellidae *A. empoascae* is known from eggs of *Empoasca* spp. on weeds, crops, and other herbaceous vegetation [29,30,31,32], so okra fits the profile of its host plants.

The five slide-mounted specimens of *A. vulneratus* from Florida differ, at least slightly, in one or more key morphological diagnostic features from all members of the *A. epos* species complex and *A. empoascae* (Table 1), as detailed below. They differ from *A. empoascae* in having fewer (2 or 3) rows of discal setae (Figure 1c) on the widest part of the fore wing (3 to 5 in *A. empoascae*); from *A. daanei*, in having 1 mps on F3 of the female antenna (Figure 1b), 3 setae on the external plate of the ovipositor (Figure 1e), and a higher (2.4–2.7) ovipositor length to fore tibia length ratio (usually no mps, almost always 2 setae, and 1.8–2.2 such ratio, respectively, in *A. daanei*); from *A. epos*, in having a relatively shorter ovipositor, with ovipositor length to fore tibia length ratio of at most 2.7 (at least 2.8 in *A. epos*); from *A. tretiakovae*, in having one longitudinal row of discal setae (Figure 1c) on the basal one-third of fore wing (two well-defined, complete such rows in *A. tretiakovae*); and from *A. vulneratus* from Colorado, in having 1 mps on F3 of the female antenna and a narrower fore wing, with length to width ratio at least 7.4 (usually no mps and at most 6.7 such ratio, respectively, in *A. vulneratus*). Thus, *A. tretiakovae* can be definitely excluded as a potentially conspecific taxon to our specimens from Florida. Among the other aforementioned species, they most closely resemble *A. empoascae* and *A. epos*, differing from them just slightly in one or more diagnostic features that are known to be prone to intraspecific variability, such as fore wing chaetotaxy and the relative length of the ovipositor [31]. Based on morphology and ecology, however, *A. vulneratus* from Florida could be conspecific with *A. empoascae*. However, a possible synonymy of *A. vulneratus* under *A. empoascae* can only be established after DNA is successfully extracted and analyzed following rearing of the latter nominal species from eggs of *Empoasca* spp. on herbaceous plants in Florida, Puerto Rico, or Haiti, within its native range.

**Figure 3:**
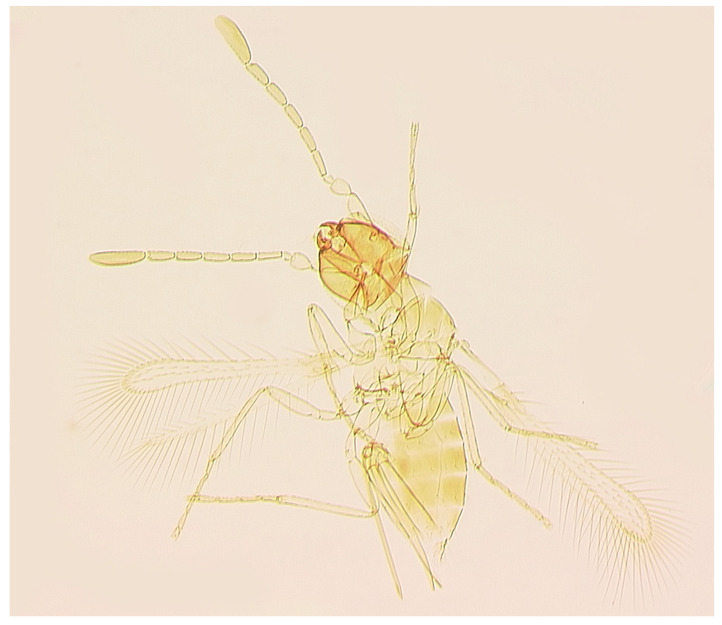
*Anagrus* sp. near *vulneratus* from Homestead, FL, USA (female): habitus (body length: 615 μm).

***Anagrus* sp. near *vulneratus* Triapitsyn** (Figure 3).

*Anagrus* sp. near *vulneratus* Triapitsyn in Triapitsyn et al. [32]: 13–14 [known from Sonora and Baja California, Mexico from eggs of *Erasmoneura variabilis* (Beamer) (Hemiptera: Cicadellidae) on cultivated grapes].

Material examined. USA: Florida, Miami-Dade Co., Homestead, UF/IFAS Tropical Research and Education Center, 25°30′36″ N 80°30′22″ W, 2 m, x.2025, Y. Velazquez-Hernandez, on okra leaves infested with *Am. biguttula* (1 female, UCRC [PR25-794; UCRC ENT 496718]).

Diagnosis: Both molecularly (Figure 2) and morphologically (Table 1), this specimen from Florida matches those of the undescribed species, *Anagrus* sp. near *vulneratus*, from Sonora, Mexico [32]. Once more specimens are collected in Florida and its molecular comparison with *A. empoascae* becomes available, a description of a new species would be warranted.

### 3.2. Molecular Identification

Variation in the COI sequences of the five Florida specimens indicated that two species were in fact present, with one specimen (PR25-794) being 3.5% different from the other four (PR25-795, PR25-796, PR25-797, and PR25-798). The same split was evident in the ITS2 sequences. Despite this divergence, initial comparison of COI against BOLD suggested that all five specimens were *Anagrus daanei* Triapitsyn, each having a similarity of >99.8% to BIN (Barcode Index Number) AAN8044. This was somewhat surprising but closer inspection of the summary data for BIN:AAN0084 (https://portal.boldsystems.org/bin/BOLD:AAN8044; accessed February 27, 2026) suggested that it may contain multiple misidentified and/or cryptic species; among almost 3000 records, average pairwise distance (a key indicator of genetic variability) was 4%, with a maximum distance in excess of 8%. In contrast, comparison of ITS2 sequences with GenBank indicated that PR25-794 was a 99.43% match for an undetermined species [FJ861063], collected from Sonora, Mexico, that Triapitsyn et al. [32] previously referred to as *A.* sp. near *vulneratus* based on its similar morphology to *A. vulneratus*. The closest matches for the remaining specimens (94.69–95.88%) were in fact a series of cloned ITS2 sequences from two *A. vulneratus* specimens [FJ861041-045], collected from Mesa Co., Colorado, and sequenced as part of the same study by Triapitsyn et al. [32].

Since the COI gene had not been sequenced by Triapitsyn et al. [32], we subsequently generated sequences for *A.* sp. near *vulneratus* and *A. vulneratus*. These were combined with the Florida sequences along with those of several other species, including two published sequences from specimens collected from grapevines in California and identified as *A. daanei* by SVT [42]. Examination of genealogical relationships among these COI sequences provided clear evidence that one was indeed *A.* sp. near *vulneratus* (uncorrected p-distance = 0.0038), and the others were almost certainly *A. vulneratus* (uncorrected p-distance = 0.0082; Figure 2). Both species were >3% different from Californian *A. daanei* (uncorrected p-distances were 0.0316 and 0.0354, respectively).

## 4. Discussion

The recent establishment of *Am. biguttula* in parts of southeastern USA and some Caribbean islands as well as in parts of Central America has increased concerns about its potential impact on staple American crops [9,10,11,43]. Additionally, since this pest is regulated and known to have resistance to pesticides [14,15,16], the findings of this study provide a promising avenue for more environmentally friendly strategies. The presented data unambiguously indicate the local origin of the two *Anagrus* species reared in southern Florida from eggs of *Am. biguttula*. Both identified members of the *Anagrus epos* species complex of the nominate subgenus of this genus, *A. vulneratus* and *A.* sp. near *vulneratus*, are not known to occur in the Old World, the origin of this leafhopper pest. Rather, they are native to North America [31,32,36] while *A. empoascae* is known throughout the New World and the Hawaiian Islands [29,30,31]. Thus, it is possible that these two species have the ability to switch from unknown local hosts in southern Florida to parasitize abundant eggs of the invasive *Am. biguttula*. We can speculate that *Empoasca* spp. could very well be the native hosts of these two parasitoid species since they and *Am. biguttula* belong to the same tribe Empoascini of the leafhopper subfamily Typhlocybinae.

Due to the presence of at least slight morphological differences from all the named members of the *A. epos* species complex (Table 1) and limited knowledge of their intraspecific variability, we must rely on unambiguous molecular data to provide these identifications. Therefore, in the meantime, it is prudent to refer to one as *A. vulneratus* and the other as *A.* sp. near *vulneratus*, until comparative COI barcode and ITS2 sequences are obtained from freshly collected specimens that fit the current morphological concept of *A. empoascae* and its type specimens from Haiti, as redescribed by Triapitsyn [29], preferably reared from *Empoasca* spp. eggs on herbaceous vegetation. Thus, additional research would be necessary to reveal the true identity of these parasitoid wasps that may be important for providing at least partial biological control of the invasive *Am. biguttula* in Florida and other southeastern states in the USA and elsewhere in the New World.

Currently, molecular identification of Mymaridae to species level using available sequences in public DNA reference databases, such as BOLD Systems for COI barcodes, needs to be approached with great caution because of a very limited accuracy and unreliability, with less than 1% of species matching in Québec, Canada [44]. Moreover, out of the two of their species that were presumed to be correctly identified based on DNA barcoding results available from BOLD Systems [44], one (*A. daanei*) was actually also misidentified, as it is not conspecific with the true *A. daanei* reared from Western grape leafhopper (*Erythroneura elegantula* Osborn, Cicadellidae; PR16-029) and Virginia creeper leafhopper (*Erythroneura ziczac* Walsh; PR16-041) eggs in California, of which we have obtained COI barcodes (Figure 2), and elsewhere. However, some of their specimens from Québec, identified as *Anagrus* sp., seem to belong to *A. daanei* based on our COI barcode data (Figure 2). Thus, an integrative taxonomy approach to the identification of these minute and speciose parasitoid wasps is essential, when feasible, in combination with building reliable genetic reference databases for as many positively recognized species as possible.

Mymarid egg parasitoids can play a pivotal role in the regulation of *Am. biguttula* populations because they can attack concealed *Am. biguttula* eggs, hence preventing leafhopper population buildup [21,22,23]. We currently lack information on the parasitism rate of *Am. biguttula* eggs by *A. vulneratus* and *A.* sp. near *vulneratus*. Future research can evaluate their efficiency as natural enemies of this leafhopper pest and shed more light on their compatibility with other IPM practices, such as chemical and cultural control. Regardless, the presence of two natural enemies that have been found responding to the invasive *Am. biguttula* can pave the way for a more sustainable management, which may also include efforts to implement a classical biological control program.

## Figures and Tables

**Table 1:** Key diagnostic characteristics in the *Anagrus epos* species complex (excluding *Anagrus tretiakovae*, after Triapitsyn et al. [32] and Triapitsyn [31]), including *Anagrus empoascae* (after Triapitsyn [31]) and *Anagrus* sp. near *vulneratus* from southern Florida. Rare occurrences are indicated in parentheses.

*Anagrus* Species	Number of mps on F3 of the Female Antenna	Fore Wing Length to Width Ratio	Number of Rows of Setae on the Widest Part of Fore Wing	Ovipositor Length to Fore Tibia Length Ratio	Number of Setae on the External Plate of Ovipositor
*A. daanei*	0 (1)	7.2–7.9	2 (3)	1.8–2.2	2 (3)
*A. empoascae*	1	7.8–8.7	3–5	2.2–2.4	3
*A. epos*	1 (0)	7.9–8.6	1–3	2.8–3.1	3
*A. vulneratus* (Colorado)	0 (1)	6.3–6.7	2–4	2.5–2.7	2–3
*A. vulneratus* (Florida)	1	7.4–9.0	2–3	2.4–2.7	3
*A.* sp. near *vulneratus* (Florida)	1	8.5	2	2.7	3
*A.* sp. near *vulneratus* (Sonora)	0–1	7.4–8.5	2–4	2.4–2.8	3

F—funicle segment of the female antenna or flagellomere of the male antenna; mps—multiporous plate sensillum or sensilla on the antennal flagellar segments.

## Data Availability

The original contributions presented in this study are included in the article. Further inquiries can be directed to the corresponding author.

## Abbreviations

The following abbreviations are used in this manuscript:

COI	Cytochrome c oxidase subunit I
ITS2	Internal transcribed spacer 2
rRNA	Ribosomal RNA
F	Funicle segment of the female antenna or flagellomere of the male antenna
mps	Multiporous plate sensillum or sensilla on the antennal flagellar segments
